# Impact of a high-fibre diet on genetic parameters of production traits in growing pigs

**DOI:** 10.1017/S1751731120001275

**Published:** 2020-11

**Authors:** V. Déru, A. Bouquet, C. Hassenfratz, B. Blanchet, C. Carillier-Jacquin, H. Gilbert

**Affiliations:** 1 GenPhySE, Université de Toulouse, INRAE, ENVT, F-31326, Castanet Tolosan, France; 2 France Génétique Porc, 35651 Le Rheu Cedex, France; 3 IFIP-Institut du Porc, 35651 Le Rheu Cedex, France; 4 UEPR, Domaine de la Prise, 35590 Saint-Gilles, France

**Keywords:** dietary fibres, feed efficiency, genetic variance, genetic by feed interactions, selection decisions

## Abstract

The use of diets with increased fibre content from alternative feedstuffs less digestible for pigs is a solution considered to limit the impact of increased feed costs on pig production. This study aimed at determining the impact of an alternative diet on genetic parameters for growth, feed efficiency, carcass composition and meat quality traits. A total of 783 Large White pigs were fed a high-fibre (**HF**) diet and 880 of their sibs were fed a conventional (**CO**) cereal-based diet. Individual daily feed intake, average daily gain, feed conversion ratio and residual feed intake were recorded as well as lean meat percentage (**LMP**), carcass yield (**CY**) and meat quality traits. Pigs fed the CO diet had better performances for growth and feed efficiency than pigs fed the HF diet. They also had lower LMP and higher CY. In addition, pigs fed the CO diet had lower loin percentage and ham percentage and higher backfat percentage. No differences were observed in meat quality traits between diets, except for a* and b* values. For all traits, the genetic variances and heritability were not different between diets. Genetic correlations for traits between diets ranged between 0.80 ± 0.13 and 0.99 ± not estimable, and none were significantly different from 0.99, except for LMP. Thus, traits in both diets were considered as mainly affected by similar sets of genes in the two diets. A genetic correlation lower than 0.80 would justify redesigning the breeding scheme; however, some genetic correlations did not differ significantly from 0.80 either. Therefore, larger populations are needed for a more definitive answer regarding the design of the breeding scheme. To further evaluate selection strategies, a production index was computed within diets for the 29 sires with estimated breeding value reliability higher than 0.35. The rank correlation between indices estimated in the CO and in the HF diet was 0.72. Altogether, we concluded that limited interaction between feed and genetics could be evidenced, and based on these results there is no need to change pig selection schemes to adapt to the future increased use of alternative feedstuffs in production farms.

## Implications

Cereals are the main base for pig diets in Europe. Increased volatility of cereal prices is foreseen due to climatic change and pressure on arable lands. When the crop prices rise, cheaper fibre-rich ingredients from industry by-products are used in commercial farms. Pig responses to these new diets should be evaluated to know if breeding schemes should be adapted to the use of alternative ingredients. Our results suggest limited genetic by feed interaction on major growth, feed efficiency, carcass traits and meat quality traits. Thus, no changes are recommended in breeding schemes to anticipate diets with increased fibre contents.

## Introduction

High-quality proteins for human food are essentially provided by livestock. Given the population increase, the global meat production is projected to rise by 16% from 2015 to 2025 (OECD/FAO, 2016). Meanwhile, the availability of lands to produce livestock feed faces competition with lands for human food and biofuels, and more severe climatic events occur in crop production areas. As a result, large feed price fluctuations are anticipated in the coming years. In France, feed cost represents already about two thirds of pig production costs (Gilbert *et al.*, 2015). Feeding pigs with by-products of the agri-food and biofuel industry could be a solution to reduce feed costs and to mobilize less land for animal feed. However, such ingredients generally contain dietary fibres and have low nutritional values. Dietary fibres have an important role in pigs to maintain normal physiological functions of the digestive tract (Wenk, 2001) but can be difficult to digest, especially for growing pigs. Phenotypic comparisons of growth, feed efficiency and carcass composition performances have already been reported in the literature for pigs fed with diets with different energy and dietary fibre contents (Quiniou and Noblet, 2012; Sevillano *et al.*, 2018). Only few studies reported the effect of high-fibre (**HF**) diets on meat quality traits (Arkfeld *et al.*, 2015). If feeding pigs with diets containing more dietary fibres generally impacts the phenotypic mean of most production traits, a major concern would be to identify whether a genetic by feed (**G × F**) interaction exists for those traits. This would imply changes in the ranking of animals on each trait and hence changes in selection decisions. If so, the genetic gain cumulated in nucleus farms where pigs are tested on conventional (**CO**) diets would not be entirely transferred to the production tier where feed may contain higher amounts of dietary fibres. Mauch *et al.* (2018) estimated high and positive genetic correlations between a diet high in energy and low in fibres and a diet low in energy but higher in fibres, for growth, feed efficiency and body composition traits in lines divergently selected for residual feed intake. They suggested that G × F interactions on feed efficiency traits are limited. However, parameter accuracies were low due to the limited number of tested animals (<350 pigs per diet). Godinho *et al.* (2018) determined genetic correlations for traits of 2230 three-way cross-bred pigs between two diets. The first diet was a typical USA diet with corn and soybean meal, and the other was a typical European diet with wheat and barley. High genetic correlations for average daily energy intake (**ADEI**) and protein deposition, and moderate genetic correlations for lipid deposition and residual energy intake were estimated. In the latter article, the North-American diet was reported as a high-energy and low-fibre diet and the European diet as a low-energy and high-fibre diet. Few references are thus available about G × F interactions on performance traits for pigs fed alternative energy and fibre levels from a European perspective. The objective of the present study was to evaluate the phenotypic and genetic (co)variances for a wide panel of production traits, including growth rate, feed efficiency, carcass composition and meat quality, between pigs fed a conventional European diet or a diet with increased fibre content. Genetic parameters were estimated between these traits within and across diets to determine G × F interactions and then the stability was assessed of selection decisions to feed ingredients diversity.

## Material and methods

### Experimental design

#### Animals

For this experiment, 1942 Large White (**LW**) maternal line male pigs entered the INRA UEPR – France Génétique Porc phenotyping station (Le Rheu, France) in 35 successive batches in 2017 and 2018 (1035 fed a CO dietary sequence and 907 fed an HF dietary sequence). Pigs that had no valid test due to health problem or injury during the test period were discarded from the analysis. In total, 1663 pigs were kept in the data set with 880 pigs fed a CO dietary sequence and 783 pigs fed an HF dietary sequence. Therefore, 15% and 14% of pigs were eliminated from the data set in the CO and in the HF diet, respectively. To maximize the genetic connectedness between the two sets of pigs and facilitate the estimation of genetic covariances between traits recorded under the two diets, a familial structure was organized, by preferentially testing pairs of full sibs. One of the siblings was fed the CO diet and the other one the HF diet. All pigs were issued from 171 sires representative of those used in the LW French collective breeding scheme, and each couple of full sibs came from a different dam.

#### Housing conditions and management

Piglets were born in selection farms from the breeding companies Axiom (Azay-sur-Indre, France) and Nucleus (Le Rheu, France) and were delivered at 3 weeks of age to the test station. Upon arrival, couples of full sibs were separated and allotted in pens of 14 animals. Each pen contained pigs from a unique farm. In each batch, defined as the group of pigs that arrived the same week at the station, all pigs were raised in the same room, with two pens of 14 pigs that were later fed the CO diet and two pens with 14 pigs later fed the HF diet. All piglets were offered the same diet during the post-weaning phase (from 3 to 9 weeks of age). At the end of the post-weaning phase, pigs were moved to growing–finishing pens without mixing them and started to be fed the CO or the HF dietary sequence. They remained in these pens until slaughter, that is, at a target 115 kg live weight. Each of these pens contained a single-place electronic feeder (**SPEF**) equipped with a weighing scale (Genstar; Acemo Skiold, Pontivy, France). Pigs had *ad libitum* access to feed and water at all stages of growth. When reaching slaughter weight, pigs were fasted 24 h before leaving the station to the slaughterhouse (Cooperl, Montfort-sur-Meu, France). All pigs were slaughtered in 89 different slaughter batches.

*Diets*. At the end of the post-weaning phase, the two sets of pigs were fed two-phase dietary sequences with compositions described in Supplementary Table S1. A growing type of diet was first distributed, then a 5-day transition was organized at 16 weeks of age (average pig live weights of 65 kg), and a finishing feed was provided until the end of test. The CO dietary sequence corresponded to the usual two-phase diet of the test station, which was formulated to cover pig energy and amino acids requirements. The feed formulation for the HF diet included soluble dietary fibres, with sugar beet pulp, and insoluble dietary fibres. Dietary sequences differed in net energy (**NE**), with 9.6 MJ/kg for the CO diet and 8.2 MJ/kg for the HF diet, and in NDF, with 13.90% for the CO diet and 23.95% for the HF diet. The ratio of digestible lysine and NE was kept identical between diets, to 0.94 g/MJ NE in the growing phase and to 0.81 g/MJ NE in the finishing phase.

### Recorded traits

#### Growth and feed efficiency traits

The test started when the animals reached 35 kg BW, as recorded by the SPEF weighing scales and ended at the target slaughter weight. Average daily feed intake (**DFI**) was calculated based on the SPEF feed consumption records during the test period. Considering the NE of the feed, the DFI was also expressed in megajoule per day (MJ/day) (DFI_J_). The average daily gain (**ADG**) was computed as the ratio between the BW gain and number of days on test. The feed conversion ratio (**FCR**) was calculated as the ratio between DFI or DFI_J_ and ADG and was expressed in kg/kg (FCR) or in MJ/kg (FCR_J_).

#### Carcass composition traits

Twenty-four hours after slaughter, the right carcasses were cut according to an industrial normalized cutting procedure (Walstra and Merkus, 1996). The primal cut weights were expressed relative to the half carcass weight as the belly percentage (**bellyP**), the loin percentage (**loinP**), the backfat percentage (**backfatP**), the shoulder percentage (**shoulderP**) and the ham percentage (**hamP**). Lean meat percentage (**LMP**) was calculated as presented in Saintilan *et al*. (2013) (see Supplementary Material S1). Carcass yield (**CY**) was calculated as the ratio between cold carcass weight 24 h after slaughter and live weight after fasting before departure to the slaughterhouse.

#### Meat quality traits

The day after slaughter, the ultimate pH (**upH**) was measured on the *semi-membranosus* ham muscle with a Sydel device (Fives Syleps, Lorient, France) equipped with an electrode Xerolyt 5 cm (LoT type, Mettler Toledo, Switzerland) above the hip bone. Lightness (L*), redness (a*) and yellowness (b*) of the meat were measured using a Minolta Chromameter CR300 (Minolta France, Carrières-Sur-Seine, France) on the most important part of the *gluteus superficialis* muscle.

#### Residual feed intake

Residual feed intake (**RFI**) was calculated as the difference between observed DFI and expected DFI for maintenance and production requirements (see Supplementary Material S2).

### Statistical analyses

#### Descriptive analyses

Pig performances were compared between the two diets. First, the variance homogeneity between diets was checked for each trait with a Levene test. Then the performance traits were analysed with linear mixed models using the SAS MIXED procedure ((SAS,^2013^) version 9.4; SAS Institute Inc., Cary, NC, USA), taking into account variance homogeneity between diets and significant fixed and random effects presented in Supplementary Table S2. In case of heteroscedasticity, different residual variances were considered for each diet. Finally, a student *t* test enabled testing the significance of the diet effect.

#### Genetic analyses

All recorded traits were part of the routine animal evaluation in the French LW collective population, so historical records were also available for the CO diet. Records from pigs reared since 2015 were included in the data set to improve the accuracy of variance component estimations. Thus, the performances of 1841 additional pigs were added to the set of 880 pigs fed the CO diet to consolidate the estimation. First, for each diet, the performances were analysed separately with animal linear mixed model described in Supplementary Material S3. Variance components were estimated by the restricted maximum likelihood (ReML) approach using the ASREML 3.0 software (Gilmour *et al.*, 2009).

The same fixed and random effects were considered in a two-trait linear mixed model, considering traits recorded with the two diets as different traits. Thus, the genetic correlations between traits were estimated both within and across diets. Genetic correlations were considered low between 0.00 and 0.20, moderate between 0.20 and 0.50 and high between 0.50 and 1.00, for absolute values.

Mulder and Bijma (2005) suggested to re-design breeding schemes when the genetic correlation between traits measured in different environments is lower than 0.80. To assess whether the genetic correlations between traits measured within diet were significantly different from 0.80 and from 0.99 (*P* < 0.05), two likelihood ratio tests were carried out as described in Supplementary Material S4. We tested the value of 0.99 because it was not possible to set the genetic correlation to one using the ASReml 3.0 software (Gilmour *et al.*, 2009).

#### Ranking of sires across diets

To assess the extent of G × F interactions, the rankings of sires were analysed based on breeding values predicted from univariate analyses using progeny performances recorded in each diet. Hence, a typical selection index comprising production traits was constructed based on the standardized estimated breeding values (**SEBVs**) of sires for ADG, FCR, CY, LMP and upH (see Supplementary Material S5). The rankings of sires were compared across diets using Spearman correlations between SEBVs of sires predicted within each diet for each individual trait and for the index.

Only the 29 sires with reliability of EBV higher than 0.35 were kept for analyses. The reliability of individual EBV is a function of the amount of information available for the animal, the structure of the information and the genetic parameters. Furthermore, 95% confidence intervals (**CIs**) were determined for the Spearman correlations using a bootstrap approach implemented in the spearman.ci function on R (R Core Team, 2016) with 1000 replicates.

## Results

### Comparison of performances between diets

Raw and adjusted means of all traits for each diet are presented in Table 1.

**Table 1:** Means and SDs of the raw performances, and least square means (along with their standard error) from linear mixed models for growing pigs fed the conventional (CO) or high-fibre (HF) diets

	Means (SD)		Homogenous residual variance	LS Means ± standard error		
	CO diet	HF diet	*P* value^1^	CO diet	HF diet	*P* value^2^
Growth and feed efficiency						
ADG (g/day)	1027 (86)	969 (85)	0.84	1027 ± 4	971 ± 4	<0.001
DFI (g/day)	2578 (230)	2692 (251)	0.02	2559 ± 8	2713 ± 9	<0.001
DFI_J_ (MJ/day)	24.25 (2.16)	22.08 (2.10)	0.18	24.11 ± 0.75	22.30 ± 0.81	<0.001
FCR	2.52 (0.15)	2.78 (0.18)	<0.001	2.52 ± 0.01	2.78 ± 0.01	<0.001
FCR_J_ (MJ/kg)	23.65 (1.46)	22.83 (1.50)	0.51	23.65 ± 0.06	22.81 ± 0.07	<0.001
RFI (g/day)	0.00 (120)	0.00 (139)	<0.001	–	+289.2 ± 8.3^3^	<0.001
Carcass composition						
CY (%)	78.89 (1.26)	77.46 (1.41)	<0.001	78.75 ± 0.05	77.60 ± 0.06	<0.001
BellyP (%)	12.65 (1.01)	12.70 (0.96)	0.21	12.66 ± 0.03	12.71 ± 0.04	0.26
LoinP (%)	28.37 (1.25)	28.80 (1.23)	0.62	28.33 ± 0.04	28.82 ± 0.04	<0.001
BackfatP (%)	7.48 (1.16)	6.55 (1.08)	0.07	7.48 ± 0.03	6.54 ± 0.04	<0.001
HamP (%)	24.13 (1.02)	24.40 (0.96)	0.13	24.17 ± 0.03	24.41 ± 0.04	<0.001
ShoulderP (%)	23.82 (0.96)	23.87 (0.96)	0.95	23.82 ± 0.03	23.84 ± 0.03	0.62
LMP (%)	58.17 (2.38)	59.82 (2.23)	0.08	58.36 ± 0.10	59.72 ± 0.11	<0.001
Meat quality						
upH	5.77 (0.19)	5.77 (0.19)	0.65	5.77 ± 0.01	5.78 ± 0.01	0.38
L*	48.12 (3.28)	47.47 (3.22)	0.63	48.01 ± 0.18	47.71 ± 0.19	0.11
a*	8.33 (1.93)	8.21 (1.83)	0.20	8.31 ± 0.13	8.08 ± 0.13	0.02
b*	8.99 (2.07)	8.71 (2.61)	0.91	9.00 ± 0.05	8.75 ± 0.08	0.01

ADG = average daily gain; DFI = daily feed intake; DFI_J_ = daily feed intake expressed in MJ/day; FCR = feed conversion ratio; FCR_J_ = feed conversion ratio expressed in MJ/day divided by kg/day; RFI = residual feed intake; CY = carcass yield; BellyP = belly percentage; LoinP = loin percentage; BackfatP = backfat percentage; HamP = ham percentage; ShoulderP = shoulder percentage; LMP = lean meat percentage; upH = ultimate pH 24 h after the slaughterhouse; L* = lightness of the meat; a* = redness of the meat; b* = yellowness of the meat

1 *P* values obtained for a test of Levene of homogeneity of variances.

2 *P* values obtained for a Student test between diets. The fixed and random effects are presented in Supplementary Table S2.

3 Contrast between animals fed the CO and the HF diet in the RFI equation and associated *P* value.

#### Growth and feed efficiency traits

Residual variances were homogeneous between diets for ADG, FCR_J_ and DFI_J_ (*P* > 0.18), and heterogeneous for FCR (*P* < 0.001), RFI (*P* < 0.001) and DFI (*P* = 0.02). Compared to pigs fed the HF diet, ADG was 6% higher (*P* < 0.001) for pigs fed the CO diet and DFI was 6% lower (*P* < 0.001). However, DFI_J_ was 8% higher (*P* < 0.001) in the CO group compared to the other group. The FCR was 9% lower for pigs fed the CO diet (2.52 ± 0.01) compared to the pigs fed the HF diet (2.78 ± 0.01, *P* < 0.001). However, when FCR was expressed in NE per kilogram of weight gain, pigs fed the CO diet had significantly higher FCR (23.65 ± 0.06 MJ/kg) compared to the other pigs (22.81 ± 0.07 MJ/kg, *P* < 0.001).

#### Carcass composition traits

Residual variances were homogeneous between diets for bellyP, loinP, backfatP, hamP, shoulderP and LMP (*P* ≥ 0.07) but significantly different for CY (*P* < 0.001). The LMP was 2% lower (*P* < 0.001) and CY was 1.5% higher (*P* < 0.001) for pigs fed the CO diet compared to the pigs fed the HF diet. Pigs fed the CO diet had lower loinP (*P* < 0.001), higher backfatP (*P* < 0.001) and lower hamP (*P* < 0.001) compared to the pigs fed the HF diet. However, there was no difference between diets for bellyP and shoulderP (*P* > 0.05).

#### Meat quality traits

Residual variances were homogeneous between diets for all meat quality traits (*P* > 0.20). The a* and b* values were higher (*P* = 0.02 and *P* = 0.01, respectively) for pigs fed the CO diet (8.31 ± 0.13 and 9.00 ± 0.05, respectively) compared to the pigs fed the HF diet (8.08 ± 0.13 and 8.75 ± 0.08, respectively). However, there was no significant difference between diets for upH (*P* = 0.38) and L* values (*P* = 0.11).

### Heritability, additive genetic and phenotypic variances

Heritability, genetic and residual variances estimated for each diet are presented in Table 2 for all traits.

**Table 2:** Heritability (h^2^), genetic and residual variances for traits for growing pigs fed the conventional (CO) and high-fibre (HF) diet, along with their standard error (SE)

	CO diet			HF diet		
	*h*² ± SE	Genetic variance ± SE	Residual variance ± SE	*h*² ± SE	Genetic variance ± SE	Residual variance ± SE
Growth and feed efficiency						
ADG (g/day)	0.40 ± 0.06	2639 ± 418	3444 ± 330	0.27 ± 0.11	1860 ± 773	4371 ± 685
DFI (g/day)	0.53 ± 0.06	19 437 ± 2518	14 661 ± 1887	0.36 ± 0.12	14 977 ± 5382	23 817 ± 4571
FCR (kg/kg)	0.47 ± 0.08	0.01 ± 0.00	0.01 ± 0.00	0.27 ± 0.11	0.01 ± 0.00	0.02 ± 0.00
RFI (g/day)	0.34 ± 0.05	5139 ± 892	8457 ± 733	0.41 ± 0.13	8448 ± 2765	11 877 ± 2319
Carcass composition						
Carcass yield (%)	0.41 ± 0.07	0.60 ± 0.10	0.82 ± 0.08	0.52 ± 0.12	0.93 ± 0.25	0.70 ± 0.20
BellyP (%)	0.27 ± 0.06	0.25 ± 0.06	0.69 ± 0.05	0.19 ± 0.10	0.16 ± 0.09	0.69 ± 0.08
LoinP (%)	0.39 ± 0.06	0.62 ± 0.10	0.98 ± 0.08	0.23 ± 0.11	0.33 ± 0.16	1.09 ± 0.14
BackfatP (%)	0.58 ± 0.06	0.77 ± 0.10	0.56 ± 0.07	0.54 ± 0.13	0.55 ± 0.15	0.47 ± 0.12
HamP (%)	0.45 ± 0.06	0.43 ± 0.07	0.52 ± 0.05	0.34 ± 0.12	0.28 ± 0.10	0.55 ± 0.09
ShoulderP (%)	0.27 ± 0.05	0.23 ± 0.05	0.64 ± 0.04	0.47 ± 0.13	0.39 ± 0.12	0.45 ± 0.09
LMP (%)	0.62 ± 0.06	3.42 ± 0.42	2.04 ± 0.31	0.56 ± 0.13	2.63 ± 0.69	1.65 ± 0.54
Meat quality						
upH	0.21 ± 0.09	0.01 ± 0.00	0.03 ± 0.00	0.16 ± 0.11	0.00 ± 0.00	0.03 ± 0.00
L*	0.21 ± 0.05	2.06 ± 0.50	7.54 ± 0.46	0.14 ± 0.10	1.36 ± 0.94	7.80 ± 0.92
a*	0.30 ± 0.05	0.84 ± 0.16	1.86 ± 0.14	0.44 ± 0.12	1.06 ± 0.32	1.29 ± 0.27
b*	0.20 ± 0.05	0.45 ± 0.11	1.71 ± 0.10	0.08 ± 0.09	0.17 ± 0.18	1.75 ± 0.18

See Table 1 for trait names.

#### Growth and feed efficiency traits

Estimated heritability ranged from 0.34 to 0.53 for ADG, FCR, DFI and RFI in the CO diet and from 0.27 to 0.41 in the HF diet. Genetic variances and heritability were not significantly different between diets for these traits.

#### Carcass composition traits

Estimated heritability for LMP was high and similar for pigs fed the CO diet and the HF diet. Estimated heritability for CY was moderate in both diets. Heritability estimates for primal cut proportions were moderate and close between diets, ranging from 0.27 to 0.58 for pigs fed the CO diet and from 0.19 to 0.54 for pigs fed the HF diet. For carcass composition traits, there was no systematic increase in genetic or residual variance with one or the other diet.

#### Meat quality traits

Heritability estimates were close between diets for upH, L*, a* and b* values. For meat quality traits, there was no systematic increase in genetic or residual variance with one or the other diet, except for a* value. For this trait, the residual variance was higher with the CO diet (1.86 ± 0.14) compared to that with the HF diet (1.29 ± 0.27).

### Genetic correlations and ranking of sires

#### Genetic correlations

Genetic correlations estimated between traits within each diet and across diets for a given trait are reported in Table 3 for all traits.

**Table 3:** Genetic correlations between traits for growing pigs fed the conventional diet (above the diagonal), fed the high-fibre diet (below the diagonal) and genetic correlations between traits across diets (on the diagonal)

Traits	ADG	DFI	FCR	RFI	CY	ShoulderP	BellyP	LoinP	BackfatP	HamP	LMP	upH	L*	a*	b*
ADG	**0.99** ^1^	0.73 ±0.05	−0.16 ±0.10	0.21 ±0.12	−0.13 ±0.12	−0.16 ±0.13	0.26 ±0.12	−0.26 ±0.11	0.42 ±0.09	−0.15 ±0.11	−0.36 ±0.09	0.30 ±0.14	0.12 ±0.14	−0.09 ±0.12	0.05 ±0.14
DFI	0.80 ±0.12	**0.96** **±0.08**	0.69 ±0.06	0.77 ±0.05	0.06 ±0.10	−0.20 ±0.06	0.47 ±0.10	−0.56 ±0.08	0.69 ±0.05	−0.44 ±0.09	−0.81 ±0.03	0.31 ±0.13	0.14 ±0.07	0.09 ±0.06	0.04 ±0.13
FCR	−0.15 ±0.28	0.54 ±0.20	**0.99** ^1^	0.72 ±0.05	0.11 ±0.11	0.09 ±0.13	0.48 ±0.12	−0.59 ±0.09	0.59 ±0.07	−0.52 ±0.09	−0.67 ±0.07	0.17 ±0.14	0.02 ±0.14	0.11 ±0.12	−0.02 ±0.14
RFI	0.27 ±0.23	0.80 ±0.09	0.68 ±0.13	**0.99** ^1^	−0.04 ±0.12	−0.09 ±0.13	0.20 ±0.14	−0.21 ±0.12	0.20 ±0.10	−0.19 ±0.12	−0.24 ±0.10	0.20 ±0.14	−0.04 ±0.07	0.02 ±0.13	−0.07 ±0.14
CY	−0.60 ±0.20	−0.37 ±0.22	0.26 ±0.23	0.01 ±0.20	**0.80** **±0.13**	−0.03 ±0.13	−0.22 ±0.13	0.05 ±0.11	−0.06 ±0.10	0.16 ±0.11	−0.96 ±0.02	−0.28 ±0.14	0.26 ±0.14	−0.05 ±0.12	0.14 ±0.14
ShoulderP	0.01 ±0.25	0.16 ±0.26	0.15 ±0.26	0.29 ±0.24	−0.21 ±0.20	**0.99** ^1^	−0.23 ±0.15	−0.25 ±0.12	−0.25 ±0.11	−0.18 ±0.12	0.00 ±0.11	−0.07 ±0.16	−0.08 ±0.16	0.01 ±0.14	−0.01 ±0.09
BellyP	0.17 ±0.32	−0.02 ±0.31	0.25 ±0.33	−0.35 ±0.31	0.00 ±0.27	−0.37 ±0.28	**0.99** ^1^	−0.55 ±0.09	0.46 ±0.11	−0.50 ±0.11	−0.56 ±0.09	0.21 ±0.17	0.14 ±0.16	−0.04 ±0.15	0.08 ±0.16
LoinP	0.24 ±0.31	−0.16 ±0.28	−0.60 ±0.27	0.24 ±0.29	0.17 ±0.25	−0.22 ±0.26	−0.37 ±0.28	**0.99** ^1^	−0.64 ±0.08	0.21 ±0.11	0.77 ±0.05	−0.32 ±0.14	−0.01 ±0.14	−0.12 ±0.12	−0.05 ±0.14
BackfatP	0.46 ±0.19	0.46 ±0.17	0.01 ±0.28	−0.29 ±0.22	−0.40 ±0.18	−0.48 ±0.18	−0.59 ±0.27	−0.62 ±0.20	**0.99** ^1^	−0.62 ±0.07	−0.96 ±0.01	0.23 ±0.13	0.13 ±0.12	0.12 ±0.11	0.10 ±0.13
HamP	−0.84 ±0.20	−0.81 ±0.18	−0.25 ±0.30	−0.18 ±0.24	0.59 ±0.19	−0.18 ±0.23	−0.42 ±0.30	0.81 ±0.29	−0.91 ±0.11	**0.99** **±0.11**	0.68 ±0.06	−0.10 ±0.14	−0.09 ±0.14	−0.08 ±0.12	0.05 ±0.14
LMP	−0.32 ±0.21	−0.49 ±0.15	−0.32 ±0.22	0.16 ±0.20	0.46 ±0.17	0.07 ±0.19	−0.77 ±0.19	0.90 ±0.10	−0.95 ±0.02	0.97 ±0.09	**0.94** **±0.06**	−0.26 ±0.12	−0.10 ±0.12	−0.16 ±0.11	−0.06 ±0.13
upH	0.91 ±0.44	0.52 ±0.39	0.05 ±0.46	0.46 ±0.33	−0.38 ±0.40	0.62 ±0.45	0.03 ±0.50	−0.15 ±0.45	0.08 ±0.35	−0.19 ±0.42	−0.18 ±0.34	**0.88** **±0.26**	−0.70 ±0.11	−0.29 ±0.15	−0.64 ±0.11
L*	0.72 ±0.31	−0.08 ±0.36	0.99^1^	−0.79 ±0.35	−0.06 ±0.30	−0.12 ±0.34	0.19 ±0.47	−0.58 ±0.45	0.37 ±0.32	−0.12 ±0.36	−0.34 ±0.31	−0.13 ±0.58	**0.89** **±0.27**	0.05 ±0.15	0.69 ±0.09
a*	−0.25 ±0.26	0.40 ±0.22	0.81 ±0.24	0.54 ±0.19	−0.04 ±0.20	0.10 ±0.21	0.53 ±0.28	−0.36 ±0.27	−0.28 ±0.20	0.29 ±0.24	0.11 ±0.20	0.10 ±0.38	−0.44 ±0.38	**0.99** ^1^	0.58 ±0.11
b*	0.14 ±0.45	0.14 ±0.44	−0.08 ±0.49	−0.05 ±0.33	−0.04 ±0.38	−0.01 ±0.41	0.99^1^	−0.73 ±0.71	−0.11 ±0.39	0.15 ±0.48	−0.05 ±0.39	−0.41 ±0.58	0.16 ±0.62	0.87 ±0.23	**0.99** ^1^

See Table 1 for trait names.

1 Estimated correlation and at the edge of the parameter space.

Genetic correlations estimated for each trait across diets were high, ranging from 0.80 to 0.99. Given the standard errors, none significantly differed from one, even if standard errors for some traits could not be estimated because estimates were at the edge of the parameter space. The likelihood ratio test results are presented in Table 4, for null hypotheses of genetic correlations between traits in the two diets equal to 0.80 and 0.99. Estimates significantly differed from 0.80 for ADG, FCR, RFI, backfatP and LMP. When genetic correlations were 0.99 under the null hypothesis, this hypothesis could never be rejected, except for LMP.

**Table 4:** Likelihood ratio tests between models comparing the likelihood under the null hypotheses H0 ‘the genetic correlation r_g_ is 0.80’ or ‘the genetic correlation r_g_ is 0.99’, with the maximum likelihood obtained with the estimated genetic correlation between traits for growing pigs fed with both diets, a conventional and a high-fibre diet^1^

Traits	Likelihood ratio test	
	H0: *r*_*g*_ = 0.80	H0: *r*_*g*_ = 0.99
ADG	10.58*	0.40
DFI	3.80	0.22
FCR	11.12*	0.40
RFI	8.30*	0.26
CY	0.00	0.14
ShoulderP	2.68	0.04
BellyP	2.12	0.04
LoinP	2.00	0.02
BackfatP	9.58*	0.12
HamP	3.26	0.00
LMP	4.72*	2.30*
upH	0.10	0.16
L*	0.12	0.12
a*	3.38	0.06
b*	2.48	0.08

See Table 1 for trait names.

1 *H0 rejected at *P* < 0.05 when the likelihood ratio test was higher than 3.84 under the null hypotheses H0 ‘the genetic correlation *r*_*g*_ is 0.80’ (*χ* ^2^ test with 1 df), and when the likelihood ratio test was higher than 1.92 under the null hypotheses H0 ‘the genetic correlation *r*_*g*_ is 0.99’ (at the bordure of the parameter space, the asymptotic null distribution is a mixture of a *χ* ^2^ distribution with 1 df and a Dirac (on zero), with equal weights, resulting in a threshold value divided by 2 compared to the usual distribution)

Genetic correlations within diets were very similar in the two diets. They were high between DFI, FCR and RFI, ranging from 0.69 to 0.77 with the CO diet and from 0.54 to 0.80 in the HF diet. The ADG was highly correlated with DFI (> 0.73 ± 0.05), and FCR and ADG had low negative correlations in both diets. The RFI and ADG had moderate correlations in the CO diet (0.21 ± 0.12) and in the HF diet (0.27 ± 0.23). The genetic correlations between primal cuts spanned from −0.64 to 0.46 in the CO diet and from −0.91 to 0.81 in the HF diet. Genetic correlations varied more in the HF diet, which may be due to the relatively high standard errors. Genetic correlations between backfatP and LMP were negative and high (-0.96 ± 0.01 in the CO diet and −0.95 ± 0.02 in the HF diet). The LMP was strongly correlated with loinP and hamP in both diets (≥0.68 ± 0.06). Genetic correlations between upH, L* and b* values were moderate to high in the CO diet, whereas they were of smaller magnitude with a* value. Genetic correlations estimated in the HF diet were relatively consistent with those estimated in the CO diet but were much less accurate.

In the CO diet, genetic correlations were positive and moderate between growth and feed efficiency traits on one hand, and bellyP and backfatP on the other hand (from 0.20 to 0.69), and they were negative and low to moderate with shoulderP, loinP and hamP (from −0.59 to 0.09). Genetic correlations for ADG, DFI, FCR and RFI were negative and moderate to high with LMP (from −0.81 to −0.24) and close to zero with CY (from −0.13 to 0.11). In the CO diet, correlations between growth and feed efficiency traits and meat quality traits were low (from −0.09 to 0.31), and carcass composition traits had low to moderate genetic correlations with meat quality traits (from −0.32 to 0.28). Within the HF diet, genetic correlations between groups of traits were more difficult to interpret due to the high standard errors. Nevertheless, these genetic correlations seemed to be in the same direction as those presented for the CO diet.

#### Ranking of sires across diets

The rank correlations between SEBV of sires predicted in each diet for ADG, DFI, FCR, CY, LMP and upH, as well as the selection index, are presented in Table 5 along with their 95% CI.

**Table 5:** Rank correlations of estimated breeding values for traits contained within the index and for the index based on sires records in the conventional diet and in the high-fibre diet

Item	Spearman correlation	95% CI	
		Lower	Upper
SEBV ADG	0.53	0.20	0.80
SEBV DFI	0.57	0.31	0.74
SEBV FCR	0.60	0.24	0.81
SEBV CY	0.59	0.26	0.79
SEBV LMP	0.46	0.08	0.73
SEBV upH	0.17	−0.39	0.40
INDEX	0.72	0.49	0.84

SEBV = estimated breeding values standardized by their genetic SD; see Table 1 for trait names.

The rank correlations between SEBV estimated for sires in the CO and in the HF diet were moderate for ADG, DFI, FCR, CY and LMP (from 0.46 to 0.60). The lowest rank correlation was for upH (0.17). The rank correlation estimated between the selection indices of sires was 0.72 in both diets.

The 95% CI estimated for rank correlations varied between 0.08 (LMP) and 0.81 (FCR), except for upH where the lower and upper bounds of the CI were −0.39 and 0.40, respectively. For the selection index combining the different production traits, the lower and upper bounds of the CI were 0.49 and 0.84.

## Discussion

The objective of the experiment was to determine whether feeding pigs with diets containing more dietary fibres impacts the mean of traits of interest for selection and whether it also influences selection decisions due to G × F interactions.

### Effect of a high-fibre diet on mean of traits of interest for selection

The increased fibre feed used in the present study was formulated to be as generic as possible including various types of fibres (soluble and insoluble) that can be found in a large number of farm diets to be representative of a lower quality diet that pigs would need to adjust by increasing their voluntary feed intake.

In the present study, the HF diet had a decreased energy content (−15%) compared to the CO diet. Pigs fed the HF diet had lower growth, DFI_J_ and feed efficiency performances than pigs fed the CO diet, which is consistent with the results reported by Quiniou and Noblet (2012) for cross-bred pigs fed different types of diets with increased fibre content and decreased NE, and with the results of Mauch *et al.* (2018) on lines divergently selected for RFI. In the present study, although pigs fed the HF diet had higher DFI (+6%), they also had lower daily NE intake (−8%). This result was also reported by Quiniou and Noblet (2012) based on barrows that had *ad libitum* access to different feeds presenting NE contents ranging from 8.1 MJ/kg to 11.1 MJ/kg: when the feed had an energy content of 8.1 MJ/kg, pigs showed significantly lower daily NE intakes than pigs allowed more concentrated feed. Although their DFI was larger, suggesting that pigs fed the HF diet were not able to compensate the energy content reduction by an increased voluntary feed intake. In the present study, the incorporation of sugar beet pulp in the HF feed may have accentuated the effect of fibrous compounds on satiety of pigs due to greater water-holding capacity (Bertin *et al.*, 1988). A complementary hypothesis would be a slight restriction due to increase in feeding times and more limited access to the feeders: access was usually saturated for 2 h during the peak of feeding events in the medium part of the growing period in the HF pens. A 8% dietary energy restriction in the HF group may explain the better energy efficiency, that is, lower FCR_J_ as well as the trend for reduced backfat thickness observed in our study, as already reported by Quiniou and Noblet (2012). Additional evaluations with different feeding systems should be run to conclude whether this effect is a direct effect of HF or an indirect effect related to the use of single-place automatic feeders. Consequently, an amino acid restriction could also be possible for pigs fed the HF diet, because the ratio of digestible lysine and NE was the same in both diets. According to Bikker *et al.* (1994), at low levels of protein intake, muscle is reduced relative to other carcass tissues. However, in our experiment, pigs fed the HF diet absorbed less amino acids per day, but they had higher loinP and hamP. So altogether the hypothesis that animals fed the HF diet had a deficiency of amino acids is unlikely.

The incorporation of dietary fibres induces an increase in the weight of digestive tract at slaughterhouse (Kass *et al.*, 1980), which could explain the lower CYs for pigs fed the HF diet in our results, due to more developed digestive tracts.

The HF diet had no effect on ultimate pH, as observed previously by Arkfeld *et al.* (2015). However, contrary to Arkfeld *et al.* (2015), we observed no significant effect of the HF diet on the lightness of the ham (L*) but a significant effect on a* and b* values, which could be an effect of the type of diets or the breeds tested.

### Effect of a high-fibre diet on genetic parameters

Heritability estimated within diets were generally close for the same trait, even if the residual variances could be influenced by the diet. Heritability estimated for growth and feed efficiency traits were consistent with those reported in the literature for LW pigs (Labroue *et al.*, 1996; Clutter, 2011; Saintilan *et al.*, 2013; Lopez *et al.*, 2018). In the present study, the heritability estimated for LMP was high but consistent with the estimates reported by Gilbert *et al.* (2007) and Lopez *et al.* (2018). Heritability estimates of other carcass traits were moderate to high and consistent with the range of values usually reported, as reviewed by Ciobanu *et al.* (2011). Heritability estimated in the CO and the HF diet for meat quality traits were similar to the previous estimates (Labroue *et al.*, 1996; Gilbert *et al.*, 2007, Ciobanu *et al.*, 2011, Saintilan *et al.*, 2013), except for a* value. Gilbert *et al.* (2007) reported a heritability of 0.21 for this trait in LW pigs, and heritability estimated in our results were higher, especially in the HF diet (0.44 ± 0.12). However, Gjerlaug-Enger *et al.* (2010) presented heritability ranging from 0.43 to 0.46 for a* value in Landrace and Duroc pigs, as observed in our population.

### Genetic by feed interactions

In the presence of G × F interactions, individuals or genotypes will respond differently to a set of feeds with contrasted composition. These interactions may lead to re-ranking of breeding individuals and hence influence selection responses in a breeding programme (Lynch and Walsh, 1998). Genetic and rank correlations are often reported to determine whether different breeding schemes should be adopted to maximize the genetic progress of different populations or production systems (Wakchaure *et al.*, 2016). Mulder and Bijma (2005) showed by simulation that genetic correlation estimates lower than 0.80 indicate sufficient genetic by environment interactions to justify revision of breeding schemes for the alternative environmental situation. The structure of the breeding scheme also influences the impact of genetic by environment interactions on selection response with sib testing being more affected than progeny-testing schemes (Mulder and Bijma, 2005).

#### Genetic correlations

For all traits, genetic correlations between diets were above 0.80 and statistically not different from 0.99 (except for LMP), suggesting that traits in both diets are mainly affected by the same set of genes. However, for most traits genetic correlations did not differ from 0.80 either, so larger populations would be needed for a definitive conclusion about the selection strategy to adopt in the future, given the relative weights of the different traits in a selection index.

Similar results were reported by Mauch *et al.* (2018) in a Yorkshire population for ADG, DFI, FCR, RFI and body composition traits, with high and positive genetic correlation (>0.87). With about 1100 three-way cross-bred pigs fed two diets with contrasted energy contents, Godinho *et al.* (2018) reported no G × F interactions for ADEI. Nevertheless, these authors reported lower genetic correlations for lipid deposition (0.62) and residual energy intake (0.76). In contrast, diets compared by Godinho *et al.* (2018) (corn and soybean *v*. wheat and barley) had higher digestibility than the HF diet presented in our experiment. Therefore, it is difficult to compare these differences between diets with our results. Furthermore, the study by Godinho *et al.* (2018) relied on three-way cross-bred pigs (synthetic sire line × (LW × Landrace)). The sire line and Landrace dam line used in this crossing may display different responses in lipid deposition due to a change in feed composition compared to the purebred LW dam line used in the present study.

#### Ranking of sires

The rank correlations estimated between sires SEBV across diets were moderate and, according to the estimated 95% CI, differed from one. Thus, there was a substantial re-ranking of sires for all traits across diets. The rank correlation observed between selection indices was slightly higher than the rank correlations estimated for each individual trait (0.72), but the bounds of the 95% CI still did not reach one. Thus, according to these results, the change in feed leads to some re-ranking of the sires. These rank correlations were obtained from 29 sires with the most accurate EBV for the different traits. However, the EBV accuracy remained limited due to a moderate number of pigs reared under the HF diet in this experimental design. Similar results were observed by Kearney *et al.* (2004): genetic correlations estimated for milk, fat and protein between two environments were high, but rank correlations between sires EBV across environments were moderate, mainly because bulls did not have enough daughters in one of the environment leading to EBV predicted with low accuracy. In addition, in our study, the 29 sires with the largest number of progenies, and thus the highest reliability, were generally those presenting the highest EBV. This pre-selection on breeding values may bias downwards the rank correlation because the variability of breeding values of those sires is reduced compared to the variability of breeding values expected in the population. Finally, the differences in SEBV means of the tested sires between diets were lower than 19% of the genetic SD. These differences were low and showed that the tested sires had very similar SEBV with the two diets. In addition, the mean difference in individual sire rank between diets was five, suggesting limited re-ranking. Ranking between diets was not consistent and it could be biased because we kept only the best sires with limited variability for analysis.

Although some re-rankings of sires were observed across diets likely due to the limiting amounts of information recorded within each diet, the high genetic correlations estimated in our study suggest that G × F interactions would be limited on growth, feed efficiency, carcass composition and meat quality traits. Thus, it does not seem necessary to adapt selection schemes to record performances of pigs fed alternative diets with increased fibre contents. This means that most of the genetic progress cumulated in the CO environment should be transferred when pigs have to perform under an alternative diet including more fibrous feedstuffs.

In conclusion, because of the volatility of feedstuff price (cereals, soybean), fibre-rich by-products of the agri-food industries may become more common in pig diets in the future, at least at certain periods of time. Our results indicate weak G × F interactions, so selecting animals in breeding nuclei with CO cereal-based feed should not hamper the transfer of cumulated genetic progress on feed efficiency but also on carcass and meat quality traits if diets evolve to include more dietary fibres. Hence, based on these results, it does not seem necessary to change the feeding practices at the selection level to increase the robustness of pig production to more diversified feeds in the future.

## Software and data repository resources

None of the data were deposited in an official repository.

## Supplementary material

For supplementary material accompanying this paper visit https://doi.org/10.1017/S1751731120001275. click here to view supplementary material
