# Supplementary material for: Impact of a high-fibre diet on genetic parameters of production traits in growing pigs
Source: Animal. 2020 Jun 19;14(11):2236–45. doi: 10.1017/S1751731120001275 (PMC7538339; doi:10.1017/S1751731120001275)
Supplement: Supplementary file 1 [file S1751731120001275sup001.docx]

SUPPLEMENTARY INFORMATION

*animal* journal

**Impact of a high fibre diet on genetic parameters of production traits in growing pigs**

V. Déru^1, 2^, A. Bouquet^3^, C. Hassenfratz^3^, B. Blanchet^4^, C. Carillier-Jacquin^1^ and H. Gilbert^1^

^1^ *GenPhySE, Université de Toulouse, INRAE, ENVT, F-31326, Castanet Tolosan, France*

*^2^ France Génétique Porc, 35651 Le Rheu Cedex, France*

*^3^ IFIP-Institut du Porc, 35651 Le Rheu Cedex, France*

*^4^ UEPR, Domaine de la Prise, 35590 Saint-Gilles, France*

Corresponding Author: Vanille Déru. E-mail: vanille.deru@inrae.fr

Table S1 Ingredient composition (%) of the conventional (CO) and the high fibre (HF) diets used in the experiment

|  | **Growing phase** | |  | **Finishing phase** | |
| --- | --- | --- | --- | --- | --- |
| **Ingredients (%)** | **CO diet** | **HF diet** |  | **CO diet** | **HF diet** |
| Wheat | 42.10 | 38.00 |  | 45.10 | 39.30 |
| Corn | 25.00 | 0.00 |  | 25.00 | 0.00 |
| Barley | 10.00 | 16.90 |  | 10.00 | 17.60 |
| Rapeseed meal | 6.00 | 6.00 |  | 10.00 | 9.90 |
| Sunflower meal no shelled | 3.00 | 3.00 |  | 4.80 | 3.00 |
| Soybean meal, 48% CP | 10.4 | 5.40 |  | 2.50 | 0.00 |
| Wheat bran | 0.00 | 15.00 |  | 0.00 | 15.00 |
| Soybean hulls | 0.00 | 8.00 |  | 0.00 | 8.00 |
| Beet pulp | 0.00 | 5.00 |  | 0.00 | 5.00 |
| _L-_Lys | 0.44 | 0.35 |  | 0.11 | 0.31 |
| _DL-_Met | 0.09 | 0.03 |  | 0.01 | 0.00 |
| _L-_Thr | 0.13 | 0.11 |  | 0.02 | 0.10 |
| Pure valine | 0.02 | 0.00 |  | 0.00 | 0.00 |
| Calcium carbonate | 1.40 | 1.12 |  | 0.12 | 1.01 |
| Dicalcium phosphate | 0.49 | 0.29 |  | 0.05 | 0.00 |
| NaCl | 0.40 | 0.40 |  | 0.40 | 0.40 |
| Vitamin and trace mineral mixture 0.5% | 0.40 | 0.40 |  | 0.40 | 0.40 |

Table S2 Fixed and random effects retained in linear mixed models

|  | **Fixed effects** | | | | | |  | **Random effects** | |  |
| --- | --- | --- | --- | --- | --- | --- | --- | --- | --- | --- |
|  | **Diet** | **Batch** | **Live weight at the beginning of the post weaning phase** | **Live weight at the end of the test period** | **Half carcass weight** | **Carcass weight with head** |  | **BatchXDietXPen** | **Slaughter date** | |
| Growth and feed efficiency |  |  |  |  |  |  |  |  |  | |
| ADG, g/day | X | X | X |  |  |  |  | X |  | |
| DFI, kg/day | X | X |  | X |  |  |  | X |  | |
| DFI_J_, MJ/day | X | X |  | X |  |  |  | X |  | |
| FCR | X | X | X |  |  |  |  | X |  | |
| FCR_J_, MJ/kg | X | X | X |  |  |  |  | X |  | |
| RFI, g/day | X |  |  | X |  |  |  | X |  | |
| Carcass composition |  |  |  |  |  |  |  |  |  | |
| Carcass yield, % | X | X |  |  |  | X |  | X |  | |
| BellyP, % | X | X |  |  | X |  |  |  |  | |
| LoinP, % | X | X |  |  | X |  |  |  |  | |
| BackfatP, % | X | X |  |  | X |  |  |  |  | |
| HamP, % | X | X |  |  | X |  |  |  |  | |
| ShoulderP, % | X | X |  |  | X |  |  |  |  | |
| LMP, % | X | X |  |  | X |  |  | X |  | |
| Meat quality |  |  |  |  |  |  |  |  |  | |
| upH | X | X |  |  |  | X |  | X | X | |
| L* | X | X |  |  |  | X |  | X | X | |
| a* | X | X |  |  |  | X |  | X | X | |
| b* | X | X |  |  |  | X |  | X | X | |

ADG = Average Daily Gain; DFI = Daily Feed Intake; DFI_J_ = Daily Feed Intake expressed in MJ per day; FCR = Feed Conversion Ratio; FCR_J_ = Feed Conversion Ratio expressed in MJ/day divided by kg/day; RFI = Residual Feed Intake; BellyP = Belly Percentage; LoinP = Loin Percentage; BackfatP = Backfat Percentage; HamP = Ham Percentage; ShoulderP = Shoulder Percentage; LMP = Lean Meat Percentage; upH = ultimate pH 24 hours after the slaughterhouse; L* = lightness of the meat; a* = redness of the meat; b* = yellowness of the meat

Material S1 Lean Meat Percentage calculation

Lean meat percentage (LMP) was calculated using the equation defined by Daumas (2008): LMP = 25.08 + 0.73 x (ham %) + 0.87 x (loin %) – 1.23 x (backfat %).

Material S2 Residual feed intake determination

Expected daily feed intake (DFI) was determined using a multiple linear regression of DFI on average daily gain (ADG) (requirements for growth), lean meat percentage (LMP) and carcass yield (CY) (requirements for body weight gain composition), and average metabolic body weight (AMW) (requirements for maintenance) (R Core Team, 2016). The AMW was calculated as described by Noblet *et al.* (1999):

AMW = (BWe^1.6^ - BWs^1.6^) / [1.6 x (BWe - BWs)].

AMW = average metabolic weight; BWe = body weight at the end of the test period; BWs = body weight at the start of the test period

Two fixed effects were included in the model, the diet and the batch. No significant interaction was found between diet and regression coefficients (*P* > 0.23). The following formula was obtained for residual feed intake (RFI), with a coefficient of determination R² = 0.73 for the multiple regression:

RFI = DFI - 1.5 x ADG + 36.3 x LMP - 10.5 x CY - 83.2 x AMW + 289.2 x DIET

with DFI expressed in g/day, ADG in g/day, LMP and CY in %, AMW in kg^0.60^, DIET = 0 for pigs fed the CO diet and 1 for pigs fed the HF diet.

Material S3 Linear mixed models

For each diet, performances were first analysed separately with linear mixed models: **y = Xb+ Zu + (Wn) + (Vq) + e**, where **y** is the vector of phenotypes for a given trait, **b** is the vector of fixed effects depending on the trait considered (Supplementary Table 2), **X** is an incidence matrix relating observations to fixed effects, **Z** is an incidence matrix of the additive genetic effects **u** ~ N(0, **A** σ²_u_) for the considered trait, where **A** is the pedigree relationship matrix built tracing back five generations of pedigree, **n** ~ N(0, **I** σ²_n_) is the vector of random common environment effects of the pen within diet within batch, applied to all traits except primal cuts ratios to carcass weight (Table 2), **W** is an incidence matrix relating performances to the random effect **n**, **q** ~ N(0,**I** σ²_q_) is the vector of random effects of slaughter date applied only for traits relative to meat quality, **V** is an incidence matrix relating performances to the slaughter batch effect, and **e** ~ N(0, **I** σ²_e_) is the vector of residual random effects.

Material S4 Test statistic of Likelihood ratio test

The likelihood ratio test (LRT) allows to test a constrained parametric model with a non-constrained parametric model. To test if the null hypothesis of a genetic correlation equal to 0.80 could be rejected, we recovered the logarithm of the maximum likelihood obtained with the ASREML 3.0 software (Gilmour *et al.*, 2009) when the model had a genetic correlation fixed at 0.80 (LogLC) and when the model was not constrained (LogLNC). The test statistic was the calculated as follows: LRT=2*(LogLNC-LogLC). The likelihood ratio test follows a Chi² distribution with one degree of freedom, and the critical value (ddl=1, P=0.05) is 3.84.

The same calculation as above was applied to test if we could reject the null hypothesis of a genetic correlation equal to 0.99.

Material S5 Index determination

All pig breeding companies have their own selection objective adapted to their specific needs. However, for breeding companies having operations in Europe, the relative weight given to production traits in the breeding objective is generally similar and defined from the economic values of those traits. Hence, a typical selection index comprising production traits was constructed based on the standardized estimated breeding values (SEBV) of sires for growth (ADG), feed efficiency (FCR), carcass composition (CY and LMP) and meat quality (upH) with the following equation: INDEX = 0.40 x SEBV_FCR + 0.35 x SEBV_ADG + 0.10 x SEBV_LMP + 0.10 x SEBV_CY + 0.05 x SEBV_ upH.

References S1 Supplementary references

Daumas G 2008. Taux de muscle des pièces et appréciation de la composition corporelle des carcasses. Journées Recherche Porcine 40, 61-68.

Gilmour AR, Gogel BJ, Cullis BR and Thompson R 2009. ASReml User Guide Release 3.0 VSN International Ltd, Hemel Hempstead, HP1 1ES, UK. Retrieved on 08 May 2020 from https://www.vsni.co.uk/.

Noblet J, Karège C, Dubois S and Van Milgen J 1999. Metabolic utilization of energy and maintenance requirements in growing pigs: effects of sex and genotype. Journal of Animal Science 77, 1208-1216.

R Core Team 2016. R: A language and environment for statistical computing. Retrieved on 9 Septembre 2019, from https://www.R-project.org/
